# Editorial: Molecular mechanisms in perinatal psychiatry

**DOI:** 10.3389/fpsyt.2025.1731095

**Published:** 2025-11-26

**Authors:** Graziano Pinna, Gita A. Pathak

**Affiliations:** 1 The Psychiatric Institute, Department of Psychiatry, College of Medicine, University of Illinois Chicago, Chicago, IL, United States; 2 UI Center on Depression and Resilience (UICDR), Department of Psychiatry, College of Medicine, University of Illinois Chicago, Chicago, IL, United States; 3 Center for Alcohol Research in Epigenetics (CARE), Department of Psychiatry, College of Medicine, University of Illinois Chicago, Chicago, IL, United States; 4 Institute for Genomic Health, Icahn School of Medicine at Mount Sinai, New York, NY, United States; 5 Department of Genetics and Genomic Sciences, Icahn School of Medicine at Mount Sinai, New York, NY, United States

**Keywords:** major depression with perinatal onset, biomarkers, community-based prevention, neuroactive steroids, inflammatory markers

Perinatal psychiatry stands at the intersection of neurobiology, psychosocial dynamics, and public health. As the field evolves, it has become increasingly clear that the molecular mechanisms underpinning perinatal mental health are deeply entwined with social and environmental determinants, particularly the presence or absence of support systems during pregnancy and postpartum. Beyond psychosocial influences, recent advances in biomarker discovery have begun to unravel the complex neuroendocrine and immunological pathways that shape vulnerability and resilience to perinatal depression (1–3).

At the molecular level, perinatal depression has been associated with dysregulation of the hypothalamic–pituitary–adrenal (HPA) axis (4), altered neurosteroid biosynthesis. especially deficits in allopregnanolone and related GABAergic modulators (5, 6), and inflammatory activation that can perturb neurotransmitter signaling (7). These biological alterations interact dynamically with psychosocial stressors, forming a biopsychosocial network in which chronic stress, hormonal withdrawal, and insufficient social buffering converge to precipitate depressive symptomatology. Peripheral biomarkers, including neuroactive steroids, inflammatory cytokines (e.g., IL-6, TNF-α), cortisol rhythms, and epigenetic markers of stress-responsive genes (8–10), provide measurable frameworks into these mechanisms and may ultimately guide individualized risk prediction and intervention strategies.

Against this backdrop, the integration of biological and psychosocial research has become essential to advancing perinatal psychiatry. This special feature brings together four pivotal studies that collectively deepen our understanding of how targeted psychosocial interventions can influence both the clinical course and the molecular correlates of perinatal depression. Together, they illustrate how bridging biomarker research and community-based preventive approaches can redefine the prevention and treatment of mood disorders during one of the most biologically and socially sensitive periods of a woman’s life.

## Epidemiological foundations: prevalence and risk factors

A large-scale cross-sectional study by He et al. evaluated the prevalence and determinants of prenatal depression among 4,564 pregnant individuals in Beijing. The prevalence of perinatal depressive symptoms was 4.1% based on the Edinburgh Postnatal Depression Scale, reflecting a lower rate compared to global estimates. The study identified parity, weight gain as indicated by higher BMI, and occupational status as significant predictors of depressive symptoms. These findings highlight not only biological and sociodemographic risk factors but also underscore the need for nuanced screening frameworks that integrate metabolic vulnerability with psychosocial context.

## Molecular mechanisms: neurosteroid pathways

Complementing this epidemiological perspective, Wenzel et al. investigated variability in neuroactive steroid derivatives of progesterone across different clinical profiles of perinatal depression, accounting for pre-pregnancy history of major depressive disorder and timing of depression onset. By analyzing blood samples from 98 pregnant participants during the first and second trimesters, they found that allopregnanolone, isoallopregnanolone, and pregnanolone levels were significantly elevated in those with perinatal-emergent depression. This suggests distinct neurosteroid pathways underlying different depression subtypes observed during pregnancy.

This pattern points to heterogeneous neurosteroid trajectories during pregnancy that may reflect dysfunction within the GABAergic stress-regulatory system. These findings lend neurobiological support to the notion that perinatal depression encompasses distinct phenotypes influenced by psychiatric vulnerability, hormonal transitions, and individual variability in neurosteroid biosynthetic capacity. Differential modulation of neurosteroids may underlie specific temporal and symptomatic subtypes of perinatal depression, offering potential biomarker targets for early risk stratification and tailored intervention.

## Intervention evidence: building social support and reducing postpartum depression

Moving from mechanisms to intervention, Tessema et al. conducted a cluster-randomized controlled trial across 32 health centers involving 550 pregnant women to investigate the impact of antenatal group-based psychoeducation on postnatal social support and postpartum depression. The intervention was grounded in a knowledge-based framework: by educating pregnant women and their caregivers about the multifaceted nature of postpartum depression (PPD) and its effects on mothers, infants, and families, the intervention aimed to strengthen awareness of social support needs and mobilize assistance.

The psychoeducation was delivered to pregnant women with moderate to severe depressive symptoms based on PHQ-9 scores during early pregnancy by frontline clinicians, who were trained by experienced psychiatrists. Sessions emphasized strategies for improving social support among postnatal mothers, leveraging the early pregnancy window when women may be more receptive to intervention before depressive symptoms intensify. The findings revealed significant increases in postnatal social support among those receiving the intervention, with participants demonstrating a reduced risk of PPD. The underlying premise proved effective: the more informed care recipients and informal caregivers became aware of the consequences of poor social support, the greater the assistance mothers received during the critical postpartum period.

In the second component of the trial, Tessema et al. examined factors that made the prenatal group-based psychoeducation intervention effective at preventing PPD. The intervention reduced PPD incidence from 28.9% in the control group to 7.6% in the intervention group. The study emphasized the protective role of multiple interconnected factors, including enhanced social support, partner’s emotional support, PPD literacy, and improved self-esteem. Additionally, the research identified key risk amplifiers, such as domestic workload and neonatal complications. These findings provide a roadmap for tailoring interventions to vulnerable populations.

## Future implications

These four studies illuminate a critical framework for understanding and addressing perinatal psychiatry. He et al. establish the epidemiological foundation, identifying key sociodemographic and metabolic risk factors that warrant targeted screening. Wenzel et al. deepen this understanding by revealing distinct neurosteroid signatures associated with perinatal-emergent depression, suggesting that the biological underpinnings of perinatal mood disorders are more heterogeneous than previously recognized. Building on these insights, Tessema et al. demonstrate that knowledge-based psychoeducational interventions can effectively disrupt the development of PPD by strengthening social support networks and enhancing maternal literacy about mental health risks.

Collectively, this body of work underscores a paradigm shift in perinatal psychiatry: the molecular mechanisms of depression cannot be addressed in isolation from the psychosocial context in which they emerge. As the field moves forward, integrating neurobiological screening with accessible, community-based psychosocial interventions offers a pragmatic and scalable approach to reducing the burden of perinatal mental health disorders globally.
